# Point–Prevalence of Antimicrobial–Related Potential Drug–Drug Interactions in Hospitalized Older Adults: A Multicenter Study Using Lexicomp

**DOI:** 10.3390/jcm15031163

**Published:** 2026-02-02

**Authors:** Yusuf Arslan, Esra Gürbüz, Sevil Alkan, Servan Vurucu, Yeliz Çiçek, Yusuf Özkaraman, Mustafa Deniz, Zekiye Hakseven-Karaduman, Ali İrfan Baran, Mehmet Çelik, Mehmet Reşat Ceylan, Tajdin İrdem, Fethiye Akgül, Deniz Altındağ, Şükran Sevim-Akıl, Elif Agüloğlu-Bali, Mustafa Kemal Çelen

**Affiliations:** 1 Department of Infectious Diseases and Clinical Microbiology, Batman Training and Research Hospital, Batman 72070, Turkey; dr.fethiyeakgul@gmail.com; 2 Department of Infectious Diseases and Clinical Microbiology, Van Training and Research Hospital, Van 65300, Turkey; dr.inanhazan@gmail.com (E.G.); zekiye.hakseven@gmail.com (Z.H.-K.); deniz.uluts@gmail.com (D.A.); 3 Department of Infectious Diseases and Clinical Microbiology, Faculty of Medicine, Çanakkale Onsekiz Mart University, Çanakkale 17020, Turkey; sevil.alkan@comu.edu.tr (S.A.); servanvurucu@comu.edu.tr (S.V.); 4 Department of Infectious Diseases and Clinical Microbiology, Faculty of Medicine, Istanbul Medipol University, İstanbul 34815, Turkey; dr.yelizcicek@gmail.com; 5 Department of Infectious Diseases and Clinical Microbiology, Kaş State Hospital, Antalya 07578, Turkey; yusufozkaraman14@outlook.com; 6 Department of Infectious Diseases and Clinical Microbiology, Antalya City Hospital, Antalya 07070, Turkey; drmustafadeniz@gmail.com; 7 Department of Infectious Diseases and Clinical Microbiology, Faculty of Medicine, Van Yüzüncü Yıl University, Van 65080, Turkey; a.irfanbaran@gmail.com; 8 Department of Infectious Diseases and Clinical Microbiology, Faculty of Medicine, Harran University, Şanlıurfa 63300, Turkey; dr.mcelik12@gmail.com (M.Ç.); dr.mresatceylan@gmail.com (M.R.C.); 9 Department of Infectious Diseases and Clinical Microbiology, Faculty of Medicine, Dicle University, Diyarbakır 21280, Turkey; tajdinirdem@msn.com (T.İ.); mkcelen@hotmail.com (M.K.Ç.); 10 Department of Infectious Diseases and Clinical Microbiology, Kızıltepe State Hospital, Mardin 47400, Turkey; sukransevim47@gmail.com; 11 Field Epidemiology Program, Department of Infectious Diseases and Early Warning, General Directorate of Public Health, Ankara 06050, Turkey; elifaguloglubali@gmail.com

**Keywords:** drug–drug interactions, polypharmacy, geriatric patients, antimicrobial, point prevalence

## Abstract

**Background/Objectives:** Potential drug–drug interaction (pDDI) refers to the co–administration of two or more drugs that interact with each other and may have therapeutic effects. Increasing rates of polypharmacy with age increase the risk of pDDIs in geriatric patients. This multicenter study aims to provide real–world data on the incidence of pDDI associated with antimicrobial therapy in hospitalized older adults. **Methods:** The study screened all hospitalized patients, including those aged 65 years and older. Using the Lexicomp^®^ Drug Interaction Online Database, researchers screened for pDDIs among all medications taken by patients. **Results:** 663 (24.0%) aged 65 and over were included in the study. Polypharmacy was present in 64.9%, and hyperpolypharmacy was present in 10.0% of the cases. 480 (72.4%) of the cases used antimicrobial therapy. The mean total number of drugs and antimicrobials used per case was 5.86 and 1.02, respectively. A total of 372 antimicrobial–related pDDIs were detected, and at least one antimicrobial–related pDDI was identified in 202 (42%) patients receiving antimicrobials. Ciprofloxacin (73.3%), clarithromycin (58.3%), and colistin (26.3%) had the highest numbers of D–type pDDIs. The antimicrobials with the highest incidence of X–type pDDIs were metronidazole (23.6%) and clarithromycin (8.3%), respectively. The logistic analysis found a significant association between antimicrobial–related pDDIs and an increase in the number of drugs, length of hospital stays, and ID departments. **Conclusions:** PDDI rates associated with antimicrobials, like the high pDDI rates associated with all drugs, support the literature. Therefore, strategies should be developed to reduce the risk of pDDI when prescribing antimicrobials to geriatric patients.

## 1. Introduction

Drug–drug interactions (DDIs), which are preventable, occur when two or more drugs interact with each other, leading to changes in the efficacy or toxicity of the drugs [1]. The term potential DDI (pDDI) refers to the co–administration of two or more drugs that interact with each other and may have therapeutic effects [2]. pDDI is a theoretical concept and does not represent actual interactions. The extent to which pDDIs lead to DDIs remains uncertain. DDIs can cause serious harm, ranging from prolonged hospital stays and readmissions to death. However, we do not fully know the true extent of damage caused by DDIs. Studies have shown that pDDIs affect 15% to 45% of hospitalized patients [3,4]. One of the most critical steps in preventing the potential harm of drug–drug interactions is identifying pDDIs and, when necessary, carefully monitoring the patient for clinical effects and implementing alternative treatment methods or treatment modifications. It is not possible to know or remember all pDDIs. It is crucial for physicians to use pDDI screening programs and databases to identify pDDIs and minimize potential DDI harm, especially when prescribing drugs. Technological advancements led to the development and ongoing updates of many pDDI screening programs and databases, such as the Lexicomp^®^ Drug Interaction Online Database [5].

In geriatric patients, the clinical adverse effects of pDDIs are more common due to decreased liver and kidney function, low lean body mass, and limited mobility [4,6]. Increasing rates of polypharmacy with age increase the risk of pDDIs in geriatric patients. Polypharmacy refers to the simultaneous use of 5 or more drugs [7]. Along with the increase in the prevalence of polypharmacy with age, the proportion of geriatric patients receiving polypharmacy has nearly tripled from 1994 to 2014, rising to approximately 42% [8]. The World Health Organization (WHO) predicts that by 2030, one in six people worldwide will be aged 60 or older. By 2050, the world’s population of people aged 60 years and older will double (2.1 billion). The number of persons aged 80 years or older is projected to triple between 2020 and 2050, reaching 426 million [9]. Geriatric patients are more susceptible to infections and, as a result, frequently take antimicrobial drugs [10]. Antimicrobial prescribing, including drug selection and dosage, is crucial in geriatric patients, but balancing efficacy, safety, tolerability, and the development of antimicrobial resistance is difficult in this patient population. Because of frequent antimicrobial prescriptions, antimicrobial–related pDDIs are of great importance for geriatric patients.

Systematic reviews analyzing the frequency of pDDIs in prescribed antimicrobials can help fill gaps, improve patient safety, inform policy development, and update evidence–based interventions. They can help improve adherence to treatment protocols and promote the rational use of antimicrobial drugs by supporting healthcare professionals in developing educational interventions. Antimicrobial drug use is prevalent among hospitalized patients, particularly in the geriatric patient population. Although there are many studies on pDDI, the literature on this topic in hospitalized geriatric patients is limited. In this context, our study aims to analyze antimicrobial–related pDDI in all hospitalized geriatric patients across nine centers using the Lexicomp^®^ Drug Interaction Online Database. Our study also examines the prevalence of polypharmacy, which is a significant problem in geriatric patients, the reasons for and appropriateness of antimicrobial drug use, and the effects of infectious disease consultations on pDDIs.

## 2. Materials and Methods

### 2.1. Study Design and Setting

This multicenter, cross–sectional, point–prevalence study screened all patients hospitalized at 9 centers on 16 January 2023 and included those aged 65 years and older. The centers included in the study are listed in Table S1. The study team recorded patients’ age, gender, chronic diseases, antimicrobials used, and other systemic drug data. All medications used by the participants, including antimicrobials and their indications, were determined (Tables S2 and S3). The researchers excluded data on topical, ophthalmic, and intranasal medications from the study. There is no specific reason for selecting January 16 as the study date. The study is cross–sectional. After obtaining ethical committee approval, the study day was determined to be a suitable date for all participants to collect data. The study evaluated all hospitalized patients in all the hospitals. Since there was no focus on any disease or service, there was no need to select a specific date.

### 2.2. Assessment of Antimicrobial Use

All nine centers participating in the study had research teams composed of infectious disease specialists. The researchers assessed the appropriateness of antimicrobial use by identifying the reasons for antimicrobial treatment (prophylaxis, empirical, and treatment) and diagnostic indications from hospital records and patient files. In this assessment process, they used the WHO AWare antibiotic book [11] and the Sanford Guide Antimicrobial Therapy [12] as guidelines. Researchers used incorrect indications, low or high doses, inappropriate durations, inappropriate combinations, and unnecessary or prolonged prophylaxis to define criteria for inappropriate antimicrobial use.

### 2.3. Assessment of Polypharmacy

Researchers meticulously documented all medications prescribed to patients using hospital records and patient files. Polypharmacy is the concurrent use of five or more drugs, whereas hyperpolypharmacy is the simultaneous use of ten or more drugs. Although the literature defines polypharmacy in various ways, we used the most widely accepted definition in our study [13].

### 2.4. Assessment of pDDI

All drugs administered concurrently, including antimicrobials and other co–prescribed medications, were entered into the Lexicomp^®^ Drug Interaction Online Database (https://www.uptodate.com/drug-interactions/?source=responsive_home#di-druglist, accessed on 13 January 2025) to identify and categorize pDDIs. FA, MD, MÇ, and the corresponding author (YA) performed data entry and evaluation on 13 January 2025. This approach allowed assessment of both antimicrobial–related and other clinically relevant interactions within the same prescription context. As per this database, the pDDIs were grouped accordingly based on the risk rating “Category A” (“No interaction”), “Category B” (“No action needed”), “Category C” (“Monitor therapy”), “Category D” (“Consider therapy modification”), and “Category X” (“Avoid combination). Additionally, all pDDIs are rated according to the criteria of “Severity” (“Minor,” “Major,” and “Moderate”) and “Reliability Rating” (“Poor,” “Fair,” “Good,” and “Excellent”). More comprehensive information on risk ratings is presented in Table S4. We chose this database for its user–friendly interface and reliability in detecting drug interactions. The database covers various drugs relevant to the study population, and it offers a paid subscription service [5].

### 2.5. Statistical Analysis

Categorical data are presented as frequencies or ratios. The normality of the data distribution was tested with a Kolmogorov–Smirnov test. Continuous data are presented as means and standard deviations or medians and ranges. As the normal distribution was not shown, the Mann–Whitney U and Kruskal–Wallis tests were used to evaluate differences in continuous variables. Differences between categorical variables were evaluated using the chi–squared test. Because the normal distribution was not shown, the correlation analyses were conducted using Spearman’s correlation test. Multiple logistic regression analyses were performed to investigate risk factors associated with pDDIs. The odds ratio (OR) and 95% confidence interval (CI) were calculated for each variable. A *p*–value of less than 0.05 was deemed significant. Statistical analysis was performed with SPSS 27.0.

### 2.6. Ethical Approval

The study complied with the Declaration of Helsinki and was approved by the Clinical Research Ethics Committee of Harran University (E–76244175–050.04.04–96803).

## 3. Results

### 3.1. Demographic Information

Of the 2767 adult patients hospitalized in hospitals excluding pediatric clinics, 663 (24.0%) were aged 65 and over and were included in the study. There were 336 males (50.7%), and the mean age was 74.3 ± 7.4 (median: 73, min–max: 65–102) years. Of the cases, 356 (53.7%) were followed up in internal medicine departments, 137 (20.7%) in surgical departments, and 170 (25.6%) in intensive care units. Patients stayed an average of 8.6 ± 7.9 days (median: 6, from 1 to 75). Overall, 68 (10.3%) cases had no chronic diseases, while the average number of chronic diseases per patient was 1.86 ± 1.3 (median: 2, min–max: 0–7). The number of cases with three or more chronic diseases was 168 (25.3%). Cardiac diseases (*n* = 411, 62.0%), diabetes mellitus (*n* = 182, 27.5%), and lung diseases (*n* = 177, 26.7%) were the most common chronic illnesses (Table S2). Of the cases, 600 (90.5%) used three or more drugs, 430 (64.9%) used five or more drugs, and 66 (10.0%) used ten or more drugs. 480 (72.4%) of the cases used antimicrobial therapy. The mean total number of medicines and antimicrobials used per case was 5.86 ± 2.7 (median: 6, min–max: 1–17) and 1.02 ± 0.9 (median: 1, min–max: 0–7), respectively, and these rates were higher in cases admitted to the intensive care unit than in other departments (*p* < 0.001) (Table 1).

### 3.2. Antimicrobial Data

Of the 480 (72.4%) patients using antimicrobials, 57 (11.9%) were receiving prophylactic treatment, 260 (54.2%) were receiving empirical treatment, and 192 (40.0%) were receiving treatment for therapeutic purposes. Of these, 324 (67.5%) used one, 124 (25.8%) used two, and 32 (6.7%) used three or more antimicrobials. The most used antimicrobials were ceftriaxone (15.8%), piperacillin–tazobactam (15.2%), meropenem (12.5%), and moxifloxacin (10.4%). By service type, the rates of antimicrobial use were 67.1% in internal medicine, 77.4% in surgery, and 79.4% in intensive care (Table 2 and Table 3).

All researchers in the study were infectious disease and clinical microbiology specialists who evaluated the adequacy of the antimicrobials administered. The infectious disease consultation (IDC) was found in 51% of cases receiving antimicrobials. The antimicrobial treatment started during the IDC was deemed adequate. In 55% of cases without IDC (29% of all cases), antimicrobial use was inadequate. Inadequate antimicrobial use was 51.8% in surgical services. 40.6% of patients in surgical services were receiving surgical prophylaxis, and low IDC rates (34.0%) with prolonged surgical prophylaxis were the most common cause of inadequate antimicrobial use (Table 2).

### 3.3. Antimicrobial–Related pDDI Data

**Figure 1:**
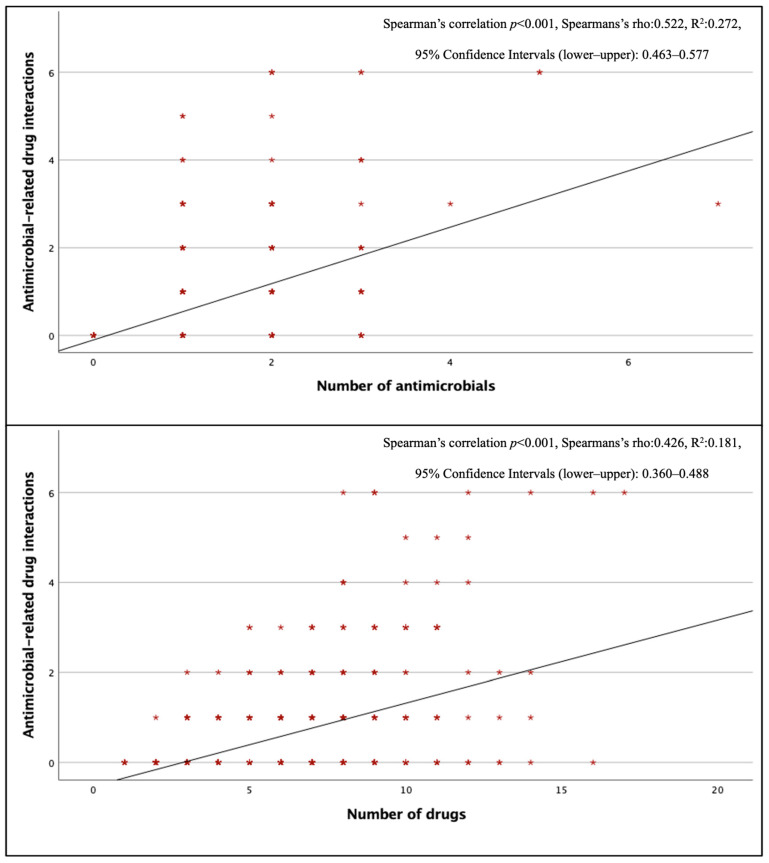
Correlation of antimicrobial–associated pDDIs with the number of antimicrobials and the number of drugs.

Three hundred seventy–two antimicrobial–related pDDIs were detected, and at least one antimicrobial–related pDDI was detected in 202 (42%) of the patients using antimicrobials. The mean number of antimicrobial–related pDDIs per case receiving antimicrobial therapy was 0.77 ± 1.21. The most common type of antimicrobial–related pDDIs was C–type (mean 0.48 ± 0.82 per case), while the mean for D–type and X–type pDDIs were 0.09 ± 0.36 and 0.02 ± 0.14, respectively. When evaluated by department, internal medicine departments had the highest frequency of antimicrobial–related pDDIs (0.98 ± 1.27 pDDIs per case). There was a positive correlation between the total number of drugs and the total number of antimicrobials and antimicrobial–related pDDIs (*p* < 0.001) (Figure 1). Looking at pDDIs related to ceftriaxone, the most frequently used antimicrobial, the mean ceftriaxone–related pDDI in cases receiving this treatment was 0.30, and ceftriaxone–related pDDIs were observed in 27.6% of cases. The average antimicrobial–related pDDI counts for the other three most used antimicrobials, piperacillin–tazobactam, meropenem, and moxifloxacin, were 0.46, 0.05, and 1.22, respectively; the rates of pDDI development were 29.7%, 4.8%, and 73.9%, respectively. Ciprofloxacin (73.3%), clarithromycin (58.3%), and colistin (26.3%) had the highest numbers of D–type pDDIs. The antimicrobials with the highest incidence of X–type pDDIs were metronidazole (23.6%) and clarithromycin (8.3%), respectively (Table 4). When looking at pDDIs between antimicrobials, there were three B–type, eight C–type, six D–type, and one X–type pDDIs. The only X–type pDDI observed among antimicrobials occurred between metronidazole and trimethoprim/sulfamethoxazole and has been associated with a disulfiram–like reaction. Type D interactions have been identified between colistin–vancomycin, colistin–amikacin, colistin–amphotericin B, and vancomycin–amikacin combinations, and these interactions have been classified as pDDIs associated with nephrotoxicity (Table S5). There was no statistically significant difference in the presence of antimicrobial–related pDDIs between groups with and without IDC (*p* = 0.394, χ^2^).

In logistic regression analysis, there was no significant difference in the occurrence of all drugs’ pDDIs regarding age, sex, number of chronic diseases, length of hospital stays, and department. PDDI was found significantly associated with an increase in the number of drugs (*p* < 0.001; adjusted odds ratio 2.885; 95% confidence interval 2.370–3.513) and antimicrobial use (*p* < 0.001; adjusted odds ratio 0.259; 95% confidence interval 0.147–0.457). There was no significant difference in the occurrence of antimicrobial–related pDDI across age, sex, or the number of chronic diseases. PDDI was found significantly associated with an increase in the number of drugs (*p* <0.001; adjusted odds ratio 1.488; 95% confidence interval 1.365–1.622), length of hospital stay for (for >14 days, *p* < 0.001; adjusted odds ratio 0.233; 95% confidence interval 0.118–0.458) and departments (for ID, *p* = 0.014; adjusted odds ratio 2.029; 95% confidence interval 1.152–3.574) (Table 5).

### 3.4. Other pDDI Information

Two thousand three hundred fifty–nine pDDIs (mean of 3.56 ± 4.68 pDDI per case) were detected with the Lexicomp^®^ Drug Interactions Online Database. The most frequently detected pDDIs were C–type pDDIs (*n* = 1641, mean 2.48 ± 3.73 pDDIs per case). The mean rates of D–type and X–type pDDIs were 0.24 ± 0.65 and 0.08 ± 0.31 per case, respectively (Table 6). One hundred thirteen patients (17.0%) had at least one D–type pDDI, 49 patients (7.4%) had at least one X–type pDDI, and 17 patients (2.6%) had both X–type and D–type pDDIs. Clopidogrel–omeprazole (*n* = 11), acetylsalicylic acid–dexketoprofen (*n* = 9), and clopidogrel–esomeprazole (*n* = 9) were the most frequent X–type pDDIs, and enoxaparin–dexketoprofen (*n* = 11), acetylsalicylic acid–diclofenac sodium (*n* = 8), and tramadol–fentanyl (*n* = 7) were the most frequent D–type pDDIs (Tables S6 and S7).

**Figure 2:**
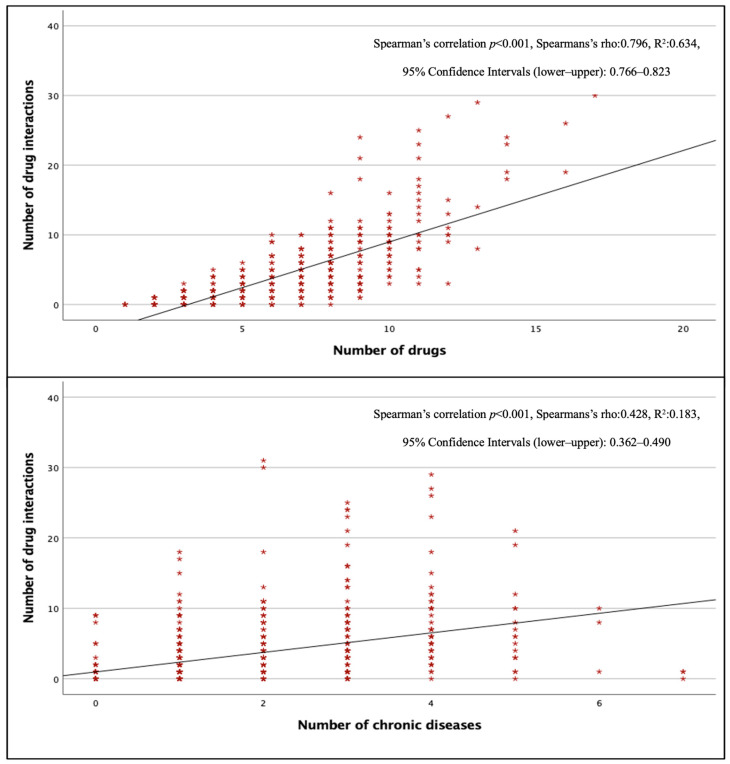
Correlation of total pDDIs with the number of drugs and the number of chronic diseases.

The total pDDI rate in surgical wards was lower than in other wards (*p* < 0.001), but there was no significant difference between internal and intensive care units (*p* > 0.05). Figure 2 shows that the number of pDDIs was strongly and positively correlated with the total number of drugs (*p* < 0.001, r = 0.796) and moderately and positively correlated with the total number of chronic diseases (*p* < 0.001, r = 0.428). The mean number of pDDIs was 3.92 ± 4.78, and the rate of pDDI was 82.2% in patients taking three or more drugs, while this number was 5.12 ± 5.14 and 93.5% in patients taking five or more drugs, and 12.36 ± 7.06 and 100% in patients taking 10 or more drugs. All patients receiving eight or more drugs had at least one pDDI.

## 4. Discussion

Globally, the geriatric population and the number of people living with multi–morbidity are increasing year by year [8,9]. With multi–morbidity, the geriatric population commonly experiences polypharmacy, using multiple medications to treat each condition [7]. The term polypharmacy denotes the simultaneous use of five or more drugs, while hyperpolypharmacy refers to the concurrent use of ten or more medications [13]. Polypharmacy is associated with adverse outcomes such as mortality, falls, adverse drug reactions, increased length of hospital stay, and readmission to the hospital immediately after discharge [14,15]. Older patients with high polypharmacy rates are at greater risk of pDDI because of reduced renal and liver function, lower lean body mass, reduced hearing, vision, cognition, and mobility [4,6]. A DDI, which is usually preventable, occurs when two or more drugs interact with each other, leading to changes in drug efficacy or toxicity [1]. Physicians and pharmacists cannot memorize all pDDIs; therefore, the use of pDDI screening programs and databases becomes even more critical [16]. Indeed, it has been shown that the use of pDDI screening programs and databases by physicians and pharmacists can reduce dangerous pDDIs by 67.5% [17].

Aging is a risk factor for infection, and antimicrobials are among the most prescribed drugs, particularly in older adults [10]. The presence of polypharmacy alongside multiple comorbidities makes optimal antimicrobial selection very difficult in geriatric patients [18]. Antimicrobial prescribing, including drug selection and dosage, is of great importance in geriatric patients; however, balancing efficacy, safety, tolerability, and the risk of antimicrobial resistance is difficult in this patient population [19]. In this study, we analyzed real–world data on pDDIs associated with commonly used antimicrobials in geriatric patients using the point–prevalence method. PDDIs were evaluated using the Lexicomp^®^ Drug Interactions Online Database. Many studies assessing the performance of pDDI screening databases report that Lexicomp^®^ Drug Interactions Online Database has high sensitivity and specificity and is often superior to other databases in these respects [16,20,21]. The selected database offers a paid subscription service because of its user–friendly interface and reliability [5]. In the database, pDDIs are categorized according to severity as A, B, C, D, and X (Table S4).

In our study, 663 geriatric patients were evaluated; polypharmacy was present in 64.9%, and hyperpolypharmacy in 10.0%. Studies report polypharmacy rates ranging from 10% to 90% [22]. One meta–analysis [23] reported a polypharmacy rate of 37% in patients aged 18 years or older, whereas another meta–analysis [13] reported a rate of 40% in geriatric patients. The first cited meta–analysis reported polypharmacy rates of 45% among individuals aged 65+ and 52% among hospitalized individuals. The prevalence of polypharmacy in older adults aged 65 years and older was found to be between 26.3% and 39.9% in 17 European countries and Israel [24]. Hyperpolypharmacy rates in studies also varied, ranging from 5% to 17% [25,26,27,28,29,30,31]. In one study, hyperpolypharmacy rates in hospitalized patients aged 70 and older were above 25%, which is considerably higher than rates reported in the literature [32]. A study from Japan [33] reported that hyperpolypharmacy rates increased dramatically with age, exceeding 40% in those aged 80 years or older. In another study, hyperpolypharmacy was detected in 51% of patients aged 70 and over with polypharmacy [34]. Differences in the prevalence of polypharmacy and hyperpolypharmacy across studies can be explained, in part, by variations in study settings and population characteristics, particularly age. One factor that may contribute to differences in study results is access to medication. In economically developed countries, the prevalence of excessive polypharmacy may be higher because of easier access to drugs. A meta–analysis [13] supports this claim, showing that polypharmacy rates are higher in developed countries than in developing countries.

In our study, 72% of patients had at least one antimicrobial use. We detected at least one antimicrobial–related pDDI in 42% of patients receiving antimicrobials (mean pDDI = 0.77). In a study of outpatients, in which 40% of prescriptions were antimicrobials, antimicrobial–related pDDI rates exceeded 20% [35]. In patients diagnosed with acute leukemia and multiple myeloma, 72.5% of participants had major–category antimicrobial–related pDDI [36]. According to Kuşçu et al.’s study [37], antimicrobials accounted for 26% of all pDDIs. In this study, the rates of major and contraindicated antimicrobial–related pDDIs reached 38% and 42%, respectively. Antimicrobial–related pDDIs were detected in 22.7% of patients receiving antimicrobials in this study (average 0.36 pDDIs per case), a rate lower than that reported in our study. This may be because the participants in this study were not geriatric patients, unlike in ours, and the polypharmacy rate was lower. The observed differences across studies can be attributed to variations in the populations selected, the medications used, and other factors. In our study, there was a positive correlation between the total number of drugs and the total number of antimicrobials, as well as with the rates of antimicrobial–related pDDI. A similar situation has been shown in other studies [37,38].

In our study, C–type and D–type pDDI ratios were higher in the quinolone and macrolide groups, while X–type pDDI ratios were higher in the metronidazole group. Our patients also had pDDIs between antimicrobials. For example, trimethoprim–sulfamethoxazole and metronidazole, which are frequently prescribed, were used together in one patient, and this combination is a cause of X–type pDDI (details are presented in Table 4 and Table 5). It has been emphasized that fluoroquinolones and clarithromycin, which are metabolized by cytochrome P450 (CYP) enzymes, should be used with caution and are high–risk antimicrobials for pDDI [39]. Metronidazole also has the potential to cause many pDDIs, like fluoroquinolones and macrolides. This is because many drugs metabolized by CYP450 2C9 and/or CYP3A4 isoenzymes inhibit liver metabolism [40]. One study reported that fluoroquinolones and clarithromycin accounted for 56% of all major antimicrobial–related pDDIs and 45% of moderate pDDIs [37]. Another study also attributed the vast majority of significant pDDIs to these two agents [41]. It is also known that the use of quinolones and clarithromycin in combination with statins, especially in elderly patients, can cause serious pDDIs [39,41]. In the study by Xu et al. [38], linezolid and levofloxacin were identified as the most common causes of pDDI. In this study, linezolid stands out as the high–risk antimicrobial, particularly in category X. Another study also found that pDDI occurred in 68% of cases associated with linezolid use and, more importantly, that 21% of patients had concomitant drugs contraindicated [42]. Therefore, particular attention should be paid to geriatric patients and to patients in intensive care units receiving linezolid. Linezolid was used in only five patients in our study, with D–type pDDI in two (40%). According to Kuşçu et al. [37], quinolones, triazoles, metronidazole, linezolid, and clarithromycin were major contributors to antimicrobial–related pDDIs. In this study, the pDDI definition differed because the Micromedex^®^ database was used instead of the Lexicomp^®^ Drug Interactions Online Database. According to the study, quinolones, metronidazole, linezolid, and clarithromycin were identified as the most critical and contraindicated antimicrobial agents causing pDDIs, respectively. Our research revealed a similar outcome when D– and X–type pDDIs were evaluated according to the Lexicomp^®^ Drug Interactions Online Database definition. In our study, although cephalosporins, penicillins, and carbapenems were the most frequently prescribed agents, the rates of D and X–type pDDIs associated with these agents were very low. A similar situation was observed in other studies, such as that by Kuşçu et al.

When considering all agents, we identified an average of 3.6 pDDIs per case in our study. At least one pDDI was present in 75.6% of all cases. A systematic review showed that 33% of general patients and 67% of ICU patients were at risk of pDDI in the hospital [1]. Another meta–analysis also found pDDI rates ranging from 8 to 100%, with rates in geriatric units exceeding 80% [43]. In our study, pDDI became inevitable when the total number of drugs exceeded 8, and the average pDDI per patient was above 12 in the presence of hyperpolypharmacy. Another study also found that pDDI was inevitable when the total number of drugs exceeded 6 [4].

Logistic regression analysis found a significant association between total pDDIs and an increase in the number of drugs and antimicrobial use. The analysis found a significant association between antimicrobial–related pDDIs and an increase in the number of drugs, length of hospital stays, and ID departments. As in our study, the number of medications was an independent risk factor for the development of pDDIs in many studies [37,41,44,45]. As in the study by Moghaddas et al. [45], the study by Teka et al. [41] also identified the number of drugs as the sole independent risk factor for pDDI. It is noteworthy that the findings of the study by Kuşçu et al. [37], which is most similar to ours in terms of study design, coincide with ours. This study identified the number of drugs and inpatient status in ID departments as risk factors for antimicrobial–related pDDI, similar to our study.

Our study had several limitations. First, all databases have shortcomings and are insufficient on their own to detect pDDIs. Therefore, a specialist physician is also required to make the final clinical decision [16]. Our second limitation is that our study is a real–life point prevalence study and an assessment of pDDI rates. We did not record the duration of medication use by patients or follow up on the actual development of DDIs. The number of studies reporting actual DDI rates is quite limited, and collecting such data requires long–term, prospective patient follow–up. It was not possible to conduct such an assessment in a point–prevalence study.

Despite the limitations, our study evaluated a large, multicenter cohort of geriatric patients and yielded significant results. The number of studies assessing pDDI rates, especially those related to antimicrobials, in geriatric patients is limited and has been conducted in small patient populations. One of the most distinctive features of our study is the identification of infectious disease specialists’ lack of awareness regarding pDDIs in antimicrobial prescribing or approval processes. In our country, many antimicrobial treatments administered to hospitalized patients require infectious disease consultation and approval. This means that other physicians cannot start antimicrobial therapy without the daily approval of an infectious disease specialist. The primary purpose of this consultation requirement is to prevent unnecessary costs and antimicrobial resistance by ensuring appropriate antimicrobial therapy for the pathogen. However, our study found no statistically significant difference in pDDI rates between the patient group that received infectious disease consultation and the group that did not. This situation shows that infectious disease specialists, who are effective decision–makers and responsible for antimicrobial treatment processes, disregard pDDI risks when selecting antimicrobials.

## 5. Conclusions

Our pDDI rates associated with antimicrobials, like the high pDDI rates associated with all drugs, support the literature. The lack of difference between groups prescribed broad–spectrum antimicrobials with and without an IDC suggests that infectious disease specialists also disregard pDDI risk when prescribing antimicrobials. Here, even if the antimicrobial prescribed for the pathogen is appropriate for the diagnosis, it may cause fatal outcomes for the patient. Therefore, strategies should be developed to reduce the risk of drug interactions when prescribing antimicrobials to geriatric patients. Examples of such strategies include training healthcare professionals, implementing antimicrobial stewardship programs, providing access to computerized screening programs or databases, and using alert systems that do not cause alert fatigue.

## Figures and Tables

**Table 1:** Data on age, length of hospital stays, medication treatments, and number of chronic diseases by gender and department.

Parameters	Distribution by Gender			Distribution by Department			
	Female (Median) Mean ± SD	Male (Median) Mean ± SD	*p* Value	ID (Median) Mean ± SD	SD ^a^ (Median) Mean ± SD	ICU ^b^ (Median) Mean ± SD	*p* Value *
*n* (%)	327 (49.3%)	336 (50.7%)	0.756 ^bin^	356 (53.7%)	137 (20.7%)	170 (25.6%)	<0.001 ^x^
Age (year)	(72) 74.39 ± 7.5	(73) 74.19 ± 7.2	0.876 ^z^	(73) ^a^ 74.55 ±7.4	(70) ^b^ 72.21 ± 6.6	(74) 75.41 ± 7.5	<0.001 ^k^
Length of hospital stays (days)	(6) 8.26 ± 7.6	(7) 6.85 ± 8.2	0.222 ^z^	(6) ^b^ 7.82 ± 6.5	(6) ^b^ 6.87 ± 5.4	(7) 11.47 ± 11.0	<0.001 ^k^
Number of drugs	(5) 5.67 ± 2.7	(6) 6.03 ± 2.8	0.104 ^z^	(6) ^ab^ 5.98 ± 2.6	(4) ^b^ 4.40 ± 2.4	(7) 6.77 ± 2.7	<0.001 ^k^
Number of antimicrobials	(1) 0.94 ± 0.8	(1) 1.10 ± 0.9	0.061 ^z^	(1) ^b^ 0.95 ± 0.8	(1) ^b^ 0.90 ± 0.6	(2) 1.27 ± 1.0	<0.001 ^k^
Number of chronic diseases	(2) 1.86 ± 1.3	(2) 1.87 ± 1.2	0.709 ^z^	(2) ^a^ 1.93 ± 1.2	(1) ^b^ 1.31 ± 1.0	(2) 2.16 ± 1.5	<0.001 ^k^

ID: internal department, SD: surgical department, ICU: intensive care unit; ^bin^ one–sample binomial test, ^z^ Mann–Whitney U test, ^x^ chi–squared test, ^k^ Kruskal–Wallis test. * *p* < 0.05 is statistically significant; ^a^ difference from the SD group; ^b^ difference from the ICU group.

**Table 2:** Reasons for administering antimicrobial treatments by department and their appropriateness.

Cases Treated with Antimicrobials	Total *n* (%)	ID *n* (%)	SD ^a^ *n* (%)	ICU ^b^ *n* (%)	*p* Value *
Total (*n*)	480 (72.4)	239 ^ab^ (67.1)	106 (77.4)	135 (79.4)	0.004 ^x^
Treatment	192 (40.0)	83 ^b^ (34.7)	31 ^b^ (29.2)	78 (57.8)	<0.001 ^x^
Empirical	260 (53.2)	159 ^a^ (66.6)	37 ^b^ (34.9)	67(49.6)	0.006 ^x^
Prophylaxis	57 (11.9)	9 ^a^ (3.8)	43 ^b^ (40.6)	5 (3.7)	<0.001 ^x^
IDC	244 (50.8)	110 ^b^ (46.0)	36 ^b^ (34.0)	98 (72.6)	<0.001 ^x^
Appropriate use	342 (71.2)	185 ^a^ (77.5)	49 ^b^ (48.2)	108 (80.0)	<0.001 ^x^

Abbreviations: ID: internal department, SD: surgical department, ICU: intensive care unit, IDC: infectious disease consultation; ^x^ chi–squared test, * *p* < 0.05 is statistically significant; ^a^ difference from the SD group; ^b^ difference from the ICU group. Note: In the 30 cases where multiple antimicrobial use was observed, there were multiple reasons for use (empirical + treatment: 25, empirical + prophylaxis: 4, empirical + prophylaxis + treatment: 1).

**Table 3:** Antimicrobial and antimicrobial–related pDDI data.

Antimicrobial *n* (%)	B–Type *n* (%)	C–Type *n* (%)	D–Type *n* (%)	X–Type *n* (%)	T–pDDI *n* (Av)	P–pDDI *n* (%)
Ceftriaxone *n* = 105 (15.8)	7 (6.7)	23 (21.9)	1 (1.0)	0	31 (0.30)	29 (27.6)
Piperacillin tazobactam *n* = 101 (15.2)	0	46 (45.5)	0	0	46 (0.46)	30 (29.7)
Meropenem *n* = 83 (12.5)	0	0	4 (4.8)	0	4 (0.05)	4 (4.8)
Moxifloxacin *n* = 69 (10.4)	29 (42.0)	53 (76.8)	1 (1.4)	1 (1.4)	84 (1.22)	51 (73.9)
Levofloxacin *n* = 51 (7.7)	34 (66.7)	59 (115.7)	9 (17.6)	0	102 (2.0)	47 (92.2)
Vancomycin *n* = 28 (4.2)	0	11 (39.3)	4 (14.3)	0	15 (0.54)	11 (39.3)
Cefazolin *n* = 28 (4.2)	0	3 (10.7)	0	0	3 (0.11)	3 (10.7)
Metronidazole *n* = 26 (3.9)	6 (23.1)	2 (7.7)	1 (3.8)	6 (23.6)	15 (0.58)	10 (38.5)
Ampicillin sulbactam *n* = 24 (3.6)	0	1 (4.2)	0	0	1 (0.04)	1 (4.2)
Colistin *n* = 19 (2.9)	0	0	5 (26.3)	0	5 (0.26)	3 (15.8)
Ciprofloxacin *n* = 15 (2.3)	4 (26.7)	18 (120.0)	11 (73.3)	0	33 (2.2)	13 (86.7)
Teicoplanin *n* = 14 (2.1)	0	0	0	0	0	0
Clarithromycin *n* = 12 (1.8)	6 (50.0)	10 (83.3)	7 (58.3)	1 (8.3)	24 (2.0)	10 (83.3)
Oseltamivir *n* = 11 (1.7)	1 (9.1)	0	0	0	1 (0.09)	1 (9.1)
Imipenem cilastatin *n* = 11 (1.7)	0	0	0	0	0	0
Other * *n* = 78 (11.8)	6 (7.7)	13 (16.7)	6 (7.7)	1 (1.3)	26 (0.33)	18 (23.1)
Total ** *n* = 480 (72.4)	90 (18.8)	231 (48.1)	43 (9.0)	8 (1.7)	372 (0.77)	202 (42.1)

Abbreviations: pDDI: potential drug–drug interaction, Av: average, T–pDDI: total number of pDDIs, P–pDDI: number of patients with pDDI. * Tigecycline (9), cefoperazone (7), trimethoprim–sulfamethoxazole (6), fluconazole (5), fosfomycin (5), linezolid (5), amikacin (4), cefepime (4), acyclovir (4), ceftazidime (4), gentamicin (3), micafungin (3), voriconazole (2), amoxicillin–clavulanate (2), cefixime (2), ceftazidime–avibactam (2), amphotericin B (2), caspofungin (2), itraconazole (1), posaconazole (1), polymyxin B (1), doxycycline (1), nitrofurantoin (1), ornidazole (1), azithromycin (1). ** Number of patients receiving at least one antimicrobial. Note: B–type pDDIs were present in 75 patients, and 3 of these were interactions between antimicrobials themselves. C–type pDDIs were present in 165 patients, and 8 of these were interactions between antimicrobials themselves. D–type pDDIs were present in 28 patients, and 6 of these were interactions between antimicrobials themselves. X–type pDDIs were present in 7 patients, and 1 of these was an interaction between antimicrobials themselves.

**Table 4:** Risk, severity, and reliability ratings of X– and D–type pDDIs associated with antimicrobials and potential risks.

Interaction Drugs (*n*)	Risk Rating	Severity	Reliability Rating	Potential Risk
Metronidazole–digoxin (2)	X	moderate	lowest	disulfiram–like reaction risk
Metronidazole–dexamethasone (1)	X	moderate	lowest	disulfiram–like reaction risk
Metronidazole–sertraline (1)	X	moderate	lowest	disulfiram–like reaction risk
Metronidazole–Trimethoprim/sulfamethoxazole (1)	X	moderate	lowest	disulfiram–like reaction risk
Metronidazole–lorazepam (1)	X	moderate	lowest	disulfiram–like reaction risk
Clarithromycin–tamsulosin (1)	X	moderate	intermediate	increased tamsulosin exposure
Moxifloxacin–quetiapine (1)	X	major	intermediate	increased risk of QT interval prolongation
Clarithromycin–budesonide (5)	D	moderate	highest	increased budesonide exposure
Meropenem–valproic acid (4)	D	major	intermediate	decrease concentrations of valproic acid
Ciprofloxacin–magnesium hydroxide (4)	D	moderate	highest	decrease absorption of quinolones
Levofloxacin–sucralfate (3)	D	major	highest	decrease absorption of quinolones
Levofloxacin–multivitamins/minerals with folate and iron (3)	D	major	highest	decrease absorption of quinolones
Vancomycin–colistin (3)	D	major	intermediate	increased risk of nephrotoxicity
Ciprofloxacin–sodium alginate + bicarbonate + calcium carbonate (2)	D	moderate	highest	decrease absorption of quinolones
Ciprofloxacin–zinc sulfate (2)	D	moderate	intermediate	decrease absorption of quinolones
Ciprofloxacin–multivitamins/minerals with folate and iron (2)	D	major	highest	decrease absorption of quinolones
Levofloxacin–sodium alginate + sodium bicarbonate + calcium carbonate (2)	D	moderate	highest	decrease absorption of quinolones
Ciprofloxacin–sucralfate (1)	D	major	highest	decrease absorption of quinolones
Levofloxacin–calcium carbonate (1)	D	moderate	highest	decrease absorption of quinolones
Clarithromycin–fentanyl (1)	D	major	intermediate	increase serum concentrations of fentanyl
Clarithromycin–atorvastatin (1)	D	moderate	intermediate	increase serum concentrations of atorvastatin
Colistin–amphotericin B (1)	D	major	intermediate	increased risk of nephrotoxicity
Colistin–amikacin (1)	D	major	intermediate	increased risk of nephrotoxicity and neuromuscular–blocking effects
Vancomycin–amikacin (1)	D	moderate	intermediate	increased risk of nephrotoxicity and neurotoxic effects
Linezolid–dobutamine (1)	D	moderate	intermediate	increase hypertensive effects of sympathomimetics
Linezolid–salbutamol (1)	D	moderate	intermediate	increase hypertensive effects of sympathomimetics
Fluconazole–fentanyl (1)	D	moderate	intermediate	increase serum concentrations of fentanyl
Fluconazole–warfarin (1)	D	major	highest	increase serum concentrations of vitamin K antagonists.
Ceftriaxone–calcium acetate and magnesium carbonate (1)	D	major	intermediate	increase adverse/toxic effects of ceftriaxone
Moxifloxacin–magnesium hydroxide (1)	D	moderate	highest	decrease serum concentrations of quinolones
Metronidazole–warfarin (1)	D	major	highest	increase serum concentrations of vitamin K antagonists.
Cefuroxime–pantoprazole (1)	D	moderate	intermediate	decrease absorption of cefuroxime
Posaconazole–pantoprazole (1)	D	major	intermediate	decrease serum concentrations of posaconazole

Abbreviations: pDDI: potential drug–drug interaction. Interaction mechanisms: Quinolones: In 10 of 11 interactions, decreased quinolone bioavailability led to reduced treatment efficacy and an increased risk of treatment failure. In the remaining interaction, there was a risk of QT prolongation because of an additive effect between moxifloxacin and quetiapine. Macrolides: There was potential for increased serum concentrations of other drugs because of inhibition of CYP3A4 or CYP2D6 enzymes. Metronidazole: In five cases, there was a risk of a disulfiram–like reaction through a mechanism yet to be explained. One of these risks was with trimethoprim–sulfamethoxazole. In one case, there was a risk of increased warfarin serum concentrations because of CYP2C9 enzyme inhibition. Cephalosporins: In one case, there was a risk of treatment failure due to decreased absorption (cefuroxime–pantoprazole), and in another, there was a risk of increased ceftriaxone toxicity following precipitation in the kidneys and lungs (with calcium acetate and magnesium carbonate). Antifungals: In one case, there was a risk of treatment failure due to decreased absorption (posaconazole–pantoprazole), and in others, a risk of decreased blood concentrations of other agents because of inhibition of CYP3A4 (fluconazole–fentanyl) and CYP2C9 + CYP3A4 (fluconazole–warfarin). Carbapenem: In one case, there was an increased risk of epileptic seizures due to decreased serum concentrations of valproic acid associated with meropenem. Linezolid: In two cases, there was an increased risk of hypertensive attacks because of monoamine oxidase (MAO) enzyme inhibition (with dobutamine and salbutamol). Others: All other interactions were additive effects, with antibiotics increasing the risk of nephrotoxicity and neurotoxic side effects.

**Table 5:** Determination of factors that predict the occurrence of pDDIs by logistic regression analysis.

	All pDDIs		Antimicrobial–Related pDDIs	
	OR (95% CI)	*p*–Value *	OR (95% CI)	*p*–Value *
Sex				
Female	reference			
Male	0.831 (0.517–1.336)	0.446	1.026 (0.705–1.493)	0.893
Age (years)	0.970 (0.937–1.004)	0.084	1.009 (0.984–1.035)	0.479
Length of hospital stays (days)				
0–7 days	reference			
8–14 days	1.304 (0.733–2.319)	0.366	1.219 (0.798–1.862)	0.360
>14 days	0.651 (0.288–1.473)	0.303	0.233 (0.118–0.458)	<0.001
Antimicrobial use				
No	reference			
Yes	0.259 (0.147–0.457)	<0.001		
Departments				
SD	Reference			
ID	0.994 (0.566–1.746)	0.984	2.029 (1.152–3.574)	0.014
ICU	0.830 (0.397–1.738)	0.622	1.506 (0.790–2.869)	0.214
Drugs (numbers)	2.885 (2.370–3.513)	<0.001	1.488 (1.365–1.622)	<0.001
Chronic diseases (numbers)	1.044 (0.820–1.330)	0.726	0.852 (0.722–1.004)	0.056

Abbreviations: OR: odds ratio; CI: confidence interval, pDDI: potential drug–drug interaction, ID: internal department, SD: surgical department, ICU: intensive care unit. * *p* < 0.05 is statistically significant.

**Table 6:** PDDI data for drugs administered to patients according to the Lexicomp^®^ Drug Interactions Online Database.

**All pDDIs (*n* = 663)**					
**Departments**	**B–Type** ***n* (Av)**	**C–Type** ***n* (Av)**	**D–Type** ***n* (Av)**	**X–Type** ***n* (Av)**	**Total** ***n* (Av)**
ID (*n* = 356)	326 (0.92)	933 (2.62)	82 (0.23)	25 (0.07)	1366 (3.84)
SD (*n* = 137)	64 (0.47)	196 (1.43)	33 (0.24)	8 (0.06)	301 (2.20)
ICU (*n* = 170)	116 (0.68)	512 (3.01)	42 (0.25)	22 (0.13)	692 (4.07)
Total	506 (0.76)	1641 (2.48)	157 (0.24)	55 (0.08)	2359 (3.56)
Antimicrobial–Related pDDIs * (*n* = 480)					
**Departments**	**B–type** ***n* (Av)**	**C–type** ***n* (Av)**	**D–type** ***n* (Av)**	**X–type** ***n* (Av)**	**Total** ***n* (Av)**
ID (*n* = 239)	72 (0.30)	138 (0.58)	22 (0.09)	3 (0.01)	235 (0.98)
SD (*n* =106)	3 (0.03)	24 (0.23)	5 (0.05)	0	32 (0.30)
ICU (*n* = 135)	15 (0.11)	69 (0.51)	16 (0.12)	5 (0.04)	105 (0.78)
Total	90 (0.19)	231 (0.48)	43 (0.09)	8 (0.02)	372 (0.77)

Abbreviations: pDDI: potential drug–drug interaction, ID: internal department, SD: surgical department, ICU: intensive care unit, Av: average. * The number of cases receiving antimicrobial therapy was 480, and at least one pDDI related to antimicrobials was detected in 202 (42.1%) of these cases. Note: Rates of pDDIs related to antimicrobial therapy were calculated using the number of cases receiving antimicrobial therapy.

## Data Availability

The data presented in this study are available upon request from the corresponding author.

## Supplementary Materials

The following supporting information can be downloaded at https://www.mdpi.com/article/10.3390/jcm15031163/s1, Table S1: Centers participating in the study; Table S2: Chronic disease information of the cases; Table S3: All medications and drug groups used by patients in our study; Table S4: Risk grading of potential drug–drug interactions by the Lexicomp^®^ Drug Interactions Program; Table S5: PDDIs between antimicrobials and each other; Table S6: D–Type drug–drug interactions other than antimicrobials; Table S7: X–Type drug–drug interactions other than antimicrobials.
