# Supplementary material for: Point–Prevalence of Antimicrobial–Related Potential Drug–Drug Interactions in Hospitalized Older Adults: A Multicenter Study Using Lexicomp
Source: J Clin Med. 2026 Feb 2;15(3):1163. doi: 10.3390/jcm15031163 (PMC12897734; doi:10.3390/jcm15031163)
Supplement: Supplementary file 1 [file jcm-15-01163-s001.zip › jcm-4106851-supplementary.pdf]

SUPPLEMENTARY TABLES

Table S1. Centers participating in the study.

Batman Training and Research Hospital
Dicle University Faculty of Medicine
Çanakkale Onsekiz Mart University Faculty of Medicine
Harran University Faculty of Medicine
Van Yuzuncu Yıl University Faculty of Medicine
Van Training and Research Hospital
Kızıltepe State Hospital
Cizre State Hospital
Bingöl State Hospital

Table S2. Chronic disease information of the cases

**Chronic disease**	n	%
Cardiac disease	411	62.0
Diabetes mellitus	182	27.5
Lung diseases	177	26.7
Neurological disease	142	21.4
Malignancy	99	14.9
Renal diseases	85	12.8
Liver diseases	23	3.5
Psychiatric diseases	19	2.9
Other	98	14.8
**Total number of chronic diseases**		
Absent	68	10.3
One	230	34.7
Two	197	29.7
Three	92	13.9
Four	53	8.0
Five	17	2.6
Six	3	0.5
Seven	3	0.5

Table S3. All medications and drug groups used by patients in our study

Drug group	Drugs used
Anti-hypertensives and cardiovascular system drugs	telmisartan + hydrochlorothiazide, irbesartan + hydrochlorothiazide, ranolazine, trimetazidine dihydrochloride, nebivolol, metoprolol, lercanidipine, valsartan + hydrochlorothiazide, amlodipine + valsartan, valsartan + sacubitril, olmesartan + hydrochlorothiazide, losartan + hydrochlorothiazide, ramipril, perindopril, benidipine, spironolactone, propranolol, diltiazem, torsemide, digoxin, carvedilol, furosemide, isosorbide mononitrate, indapamide, nifedipine, verapamil + trandolapril, bisoprolol, nimodipine, amiodarone, dobutamine
Anti-diabetics	metformin, empagliflozin, insulin aspart + insulin aspart protamine, insulin aspart, insulin detemir,

	linagliptin, gliclazide, pioglitazone, insulin glargine, glimepiride, vildagliptin, acarbose, exenatide, dapagliflozin, sitagliptin
Anti-inflammatories/anti-analgesics	acemetacin, etodolac, paracetamol, tramadol, fentanyl, naproxen, indomethacin, dexketoprofen, diclofenac, tenoxicam, ibuprofen, flurbiprofen, meloxicam, chlorzoxazone
Proton pump inhibitors/gastrointestinal regulators	pantoprazole, domperidone, ondansetron, esomeprazole, omeprazole, lansoprazole, metoclopramide, hyoscine-N-butylbromide, sucralfate, famotidine, granisetron
Anti-coagulants/antiaggregants	Enoxaparin, acetylsalicylic acid, clopidogrel, rivaroxaban, warfarin, apixaban, ticagrelor, dabigatran
Lipid regulators	atorvastatin, fenofibrate, rosuvastatin
Anti-depressants/anti-psychotics	sertraline, quetiapine, duloxetine, escitalopram, trazodone, olanzapine, risperidone, venlafaxine, lorazepame, midazolam, diazepam, levetiracetam
Bronchodilators	salbutamol, salbutamol + ipratropium bromide, salmeterol xinafoate + fluticasone propionate, formoterol fumarate + budesonide, montelukast, theophylline, pheniramine
Hormones	levothyroxine, megestrol, bicalutamide
Anti-rheumatic drugs	sulfasalazine, hydroxychloroquine sulfate, leflunomide
Corticosteroids/immunosuppressives/ monoclonal antibodies	methylprednisolone, methotrexate, mycophenolate mofetil, everolimus, tacrolimus, denosumab, rituximab, azathioprine, gemcitabine, docetaxel, dexamethasone, mitomycin-c, rapamune, lenalidomide, doxorubicin, vincristine, cyclophosphamide, fludarabine, Cytosine arabinoside, Idarubicin, everolimus, leflunomide, adalimumab, bortezomib, lapatinib, capecitabine, imatinib, bevacizumab,
Anti-epileptics	gabapentin, sodium valproate, levetiracetam, phenytoin, pregabalin
Urinary system and benign prostatic hypertrophy medications	darifenacin, dutasteride, alfuzosin, tamsulosin, silodosin, doxazosin
Other	allopurinol, piracetam, memantine, alendronate sodium + cholecalciferol, calcium carbonate + cholecalciferol, alpha-lipoic acid, ursodeoxycholic acid, cilostazol, colchicine, pyridostigmine, rivastigmine, filgrastim, lenograstim, dapsone, betahistine, rasagiline, pramipexole, benserazide, donepezil, methimazole, dimenhydrinate, oxycodone, haloperidol, Remifentanil, zinc sulfate, Magnesium Hydroxide, multivitamins/minerals (with ADEK, folate, iron), calcium carbonate, calcium acetate + magnesium carbonate

Table S4. Risk grading of potential drug-drug interactions by the Lexicomp® Drug Interactions Program.

Risk grading	Approach to interaction	Additional information
A	No known interactions	No pharmacodynamic or pharmacokinetic interactions have been demonstrated between the specified agents.
B	No action required	It has been demonstrated that the indicated agents may interact with each other, but there is little or no evidence of clinical concern arising from the concomitant use of these agents.
C	Follow the treatment	These agents may interact with each other. The benefits of the combined use of these two agents outweigh the risks. An appropriate monitoring plan should be in place to identify possible adverse effects. Dose adjustment of the drugs may be necessary.
D	Consider change of treatment	Clinically significant interactions between the two medicinal products have been demonstrated. Special precautions should be taken to maximize the benefits and/or minimize the risks arising from the concomitant use of the drugs. These precautions may include intensive monitoring, empirical dose changes, or preference for alternative medicines.
X	Avoid combinations	The data suggests that these agents may interact with each other in a clinically significant manner. The risks associated with the concomitant use of these agents generally outweigh the benefits. Concomitant use of these agents should generally be avoided.

Table S5. PDDIs between antimicrobials and each other

pDDI Type	Antimicrobial 1	Antimicrobial 2	n
B	Metronidazole	Levofloxacin	2
B	Metronidazole	Fluconazole	1
C	Trimethoprim/Sulfamethoxazole	Levofloxacin	3
C	Piperacillin Tazobactam	Vancomycin	2
C	Fluconazole	Levofloxacin	1
C	Gentamycin	Ceftriaxone	1
C	Gentamycin	Cefazolin	1
D	Colistin	Vancomycin	3
D	Colistin	Amphotericin B	1
D	Colistin	Amikacin	1
D	Vancomycin	Amikacin	1
X	Metronidazole	Trimethoprim/Sulfamethoxazole	1

Table S6. D-Type drug-drug interactions other than antimicrobials

Drug 1	Drug 2	n	Drug 1	Drug 2	n
enoxaparin	dexketoprofen	11	acetylsalicylic acid	acemetacin	1
acetylsalicylic acid	diclofenac	8	acetylsalicylic acid	etodolac	1
tramadol	fentanyl	7	acetylsalicylic acid	methotrexate	1
deksametazon	phenytoin	5	deksametazon	lenalidomide	1
enoxaparin	diclofenac	4	gliclazide	empagliflozin	1
tramadol	hyoscine-n-butylbromide	3	gliclazide	dapagliflozin	1
tramadol	quetiapine	3	gliclazide	metformin	1
enoxaparin	flurbiprofen	3	gliclazide	pioglitazone	1
midazolam	fentanyl	3	gliclazide	vildagliptin	1
diclofenac	enoxaparin	3	quetiapine	haloperidol	1
tramadol	diazepam	2	quetiapine	olanzapine	1
tramadol	lorazepam	2	methotrexate	etodolac	1
enoxaparin	tenoxicam	2	methotrexate	pantoprazole	1
acetylsalicylic acid	ticagrelor	2	midazolam	remifentanil	1
quetiapine	escitalopram	2	furosemide	tenoxicam	1
methotrexate	lansoprazole	2	prednizolon	leflunomide	1
furosemide	diclofenac	2	warfarin	amiodaron	1
sucralfate	furosemide	2	warfarin	diclofenac	1
tramadol	dimenhydrate	1	warfarin	meloxicam	1
tramadol	pheniramine	1	dexketoprofen	escitalopram	1
tramadol	levetiracetam	1	dexketoprofen	ibuprofen	1
tramadol	midazolam	1	hyoscine-n-butylbromide	fentanyl	1
tramadol	oxycodone	1	atorvastatin	verapamil	1
tramadol	olanzapine	1	budesonide	verapamil	1
tramadol	pregabalin	1	gliclazide	sitagliptin	1
enoxaparin	ibuprofen	1	escitalopram	quetiapine	1
enoxaparin	indomethacin	1	flurbiprofen	furosemide	1
acetylsalicylic acid	ibuprofen	1	Humulin	linagliptin	1
acetylsalicylic acid	meloxicam	1	insulin	pioglitazone	1
acetylsalicylic acid	tenoxicam	1	colchicine	diltiazem	1
lansoprazole	exenatide	1	oxycodone	lorazepam	1
Lantus	linagliptin	1	perindopril	irbesartan	1
NoVo rapid	pioglitazone	1	salbutamol	carvedilol	1

Table S7. X-Type drug-drug interactions other than antimicrobials

Drug 1	Drug 2	n
clopidogrel	omeprazole	11
acetylsalicylic acid	dexketoprofen	9
clopidogrel	esomeprazole	9
salbutamol	propranolol	3
diclofenac	tenoxicam	2
doxazosin	silodosin	2
quetiapine	metoclopramide	2

nimodipine	phenytoin	2
dexketoprofen	ibuprofen	1
valsartan	sacubitril	1
pheniramine	ipratropium	1
carvedilol	salbutamol	1
enoxaparin	rivaroxaban	1
enoxaparin	apixaban	1
salbutamol	carvedilol	1
tacrolimus	rapamune	1
